# The Association between Multiple Per- and Polyfluoroalkyl Substances’ Serum Levels and Allostatic Load

**DOI:** 10.3390/ijerph19095455

**Published:** 2022-04-29

**Authors:** Tahir Bashir, Emmanuel Obeng-Gyasi

**Affiliations:** 1 Department of Built Environment, North Carolina A&T State University, Greensboro, NC 27411, USA; tmbashir@aggies.ncat.edu; 2 Environmental Health and Disease Laboratory, North Carolina A&T State University, Greensboro, NC 27411, USA

**Keywords:** PFAS, per- and polyfluoroalkyl substances, income, ethnicity, smoking, physical activity, alcohol, allostatic load

## Abstract

***Background/Objective***: This study aimed to explore the association between allostatic load (AL), an index of chronic stress, with nine per- and polyfluoroalkyl substances (PFASs), a group of organic compounds used in commercial and industrial applications. The PFASs explored were perfluorohexane sulfonic acid (PFHS), perfluorodecanoic acid (PFDE), perfluorobutane sulfonic acid (PFBS), perfluoroheptanoic acid (PFHP), perflurododecanoic acid (PFDO), perfluorononanoic acid (PFNA), perfluoroundecanoic acid (PFUA), perfluorooctanoic acid (PFOA), and perfluorooctane sulfonic acid (PFOS). This study was performed to better understand the association between PFASs and AL, which may be a mediator of several diseases. ***Methods***: This study was performed on adults aged 20 and older, using the National Health and Nutrition Examination Survey (NHANES) 2007–2014 data. AL was calculated as a cumulative index of ten biomarkers from the cardiovascular, inflammatory, and metabolic system, which was dichotomized into high risk (assigned a value of 1) or low risk (assigned a value 0) depending on if the index value was ≥3 (chronic physiological stress) or <3 (less stressed). In this study, PFASs and covariates such as age, gender, ethnicity, alcohol consumption, smoking, and physical activity were explored using descriptive statistics and logistic regression modeling. ***Results***: The results indicated that in adults, AL was more elevated in men as compared to women, in those aged ≥60 years, and varied by ethnicity. For instance, non-Hispanic Blacks had higher AL levels (mean of 3.92) compared to other ethnicities. A significant number of the participants tested for PFBS, PFHP, PFDO were below the LOD and thus these PFASs were excluded from the analysis. Our analysis demonstrated multicollinearities between variables such as PFNA, PFOS, and PFOA with variance inflation factor (VIF) values of 6.197, 6.212, and 5.139, respectively. Thus, PFASs were analyzed individually and adjusted for age, gender, ethnicity, physical activity, smoking, and alcohol consumption. The results indicated a statistically significant positive association between AL and most of the PFASs, except PFUA which was not statistically significant with a *p* value of 0.531. ***Conclusions***: The findings of this study suggest that exposure to PFDE, PFNA, PFOS, PFOA, and PFHS are associated with AL when adjusted for age, gender, ethnicity, alcohol consumption, smoking, and physical activity. Future studies looking to model the effects of these factors together must consider their relationship with each other and choose different analytical approaches.

## 1. Introduction

Per- and polyfluoroalkyl substances (PFASs) are persistent organic pollutants that have been widely used in consumer products for more than seven decades. Because of PFASs unique thermal stability and surface activity properties, such as hydro- and lipophobicity, they have been used in commercial and industrial applications [1]. For example, PFASs have been utilized in food packaging, firefighting foams, and coatings to bring about nonstick and stain-resistant properties and are also used as lubricants in industrial processes and as additives in insecticides and pharmaceuticals [2].

More than 4700 PFASs exist, with many having different properties [3]. These properties mean that they are used in various applications; for this reason, different PFASs are prevalent in multiple settings.

The home environment, including house dust and contaminated foods (e.g., seafood), are primary sources of human exposure, with drinking water in areas of the United States (US) also found to be contaminated with PFASs [4,5]. Due to extensive exposure to PFASs from water, air, and food, in addition to the environmental and biological persistence of some PFASs, measurable levels of them can be found in the blood of a significant percentage of the population in developed and developing countries [6].

Chronic physiological stress is an unavoidable part of human life. Allostatic load (AL) provides insight into the effects of accumulated stress. The physiological response of the stress processes is to promote the adaptation of the body to changing stimuli while preserving homeostasis.

The term allostatic refers to physiological effects that are activated to achieve “stability through change”, which captures dysregulation across various biological systems such as the cardiovascular, metabolic, and inflammatory systems [7,8]. In turn, AL reflects the cost paid by the body for continual adaptation to environmental stressors [9,10]. An elevated AL is the result of excessive stress or the inadequacy of adaptive allostatic processes [8]. AL is associated with disease and dysfunction. A study by Guidi and colleagues [11] found that an increased AL in older adults alters brain function, diminishes the immune system, promotes Alzheimer’s disease, and increases the risk of death. AL elevation has also been linked with an increased risk for cardiovascular diseases and cancer [12,13]. AL is operationalized using the AL index, a cumulative index of 10 selected biomarkers of the cardiovascular, inflammatory, and metabolic systems. In many studies using AL, biomarkers are designated as either high risk or low risk depending on their distribution, with a value of 1 assigned for high risk or 0 for low risk. After summation of indices out of 10, participants with an AL ≥3 are considered as having high AL, with those <3 considered as having low AL [12,14].

**Figure 1:**
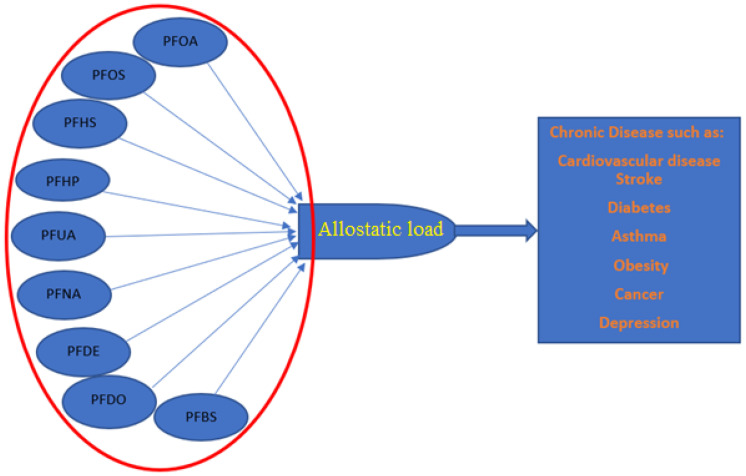
The potential relationship between PFASs and AL and the role AL may play as a mediator of chronic diseases outcomes.

This study aimed to examine the associations between chronic physiological stress, as operationalized by AL, and the serum concentration of nine PFASs using the National Health and Nutrition Examination Survey from 2007–2014. The PFASs examined were perfluorohexane sulfonic acid (PFHS), perfluorodecanoic acid (PFDE), Perfluorobutane sulfonic acid (PFBS), perfluoroheptanoic acid (PFHP), perflurododecanoic acid (PFDO), perfluorononanoic acid (PFNA), perfluoroundecanoic acid (PFUA), perfluorooctanoic acid (PFOA), and perfluorooctane sulfonic acid (PFOS). This study is critical since AL may be a mediator for several chronic diseases, such as cardiovascular disease and cancer [11], and understanding the association between AL and multiple PFASs can offer insight into how exposure to the multiple contaminants affects health. This is especially critical since real-life human exposure to pollutants is highly variable and temporally dynamic [15]. Figure 1 illustrates the role PFASs may play in elevating AL, a mediator for potential chronic disease outcomes.

## 2. Materials and Methods

### 2.1. Description of Cohort

This study analyzed de-identified demographic and biomarker data collected for the National Health and Nutrition Examination Surveys (NHANES), which is administered by the Centers for Disease Control and Prevention (CDC). NHANES uses a complex, multistage probability design that samples the civilian noninstitutionalized population residing in all 50 states within the United States and the District of Columbia (DC). The target population for this study was adults aged 20 and older.

### 2.2. Blood Serum Measurements

NHANES collected biological specimens for laboratory analysis to provide detailed information about participants’ health and nutritional status at the mobile examination center (MEC). Sera were stored in polypropylene and polyethylene containers. The blood sample collection depended on the age of the participant and collected a minimum of 0.5 mL of serum, preferably. Blood was processed, then refrigerated or frozen before being shipped to laboratories throughout the United States. The controlled environment of the MEC permitted laboratory measurements to be completed under similar conditions at each survey location.

Participants in this study appointed to a morning session were asked to fast for 9 h. After the primary blood draw, they were asked to consume 75 g of dextrose (10 oz of glucose solution) within ten minutes. A second blood sample was collected two hours later [16,17].

### 2.3. PFASs Extraction and Quantitation

The extraction of PFASs was performed. After dilution with formic acid, one aliquot of 50 μL of serum was injected into a commercial column switching system allowing for concentration of the analytes (PFASs) on a solid-phase extraction column. The NHANES followed the recommended phlebotomy practices for the collection of blood and separation of blood serum. The PFAS analysis was completed on participants’ blood sera who completed and passed the questionnaires to screen for conditions such as hemophilia, having been administered chemotherapy in the previous four weeks, and other reasons that would preclude using the participant’s arms for a blood draw [18,19].

Partitioning of the analytes from each other and other serum components was performed with high-performance liquid chromatography. Detection and quantification were done using a negative-ion Turbo Ion Spray (TIS) ionization source, which is a variant of the electrospray ionization source that is used to convert liquid phase ions into gas-phase ions incorporated into tandem mass spectrometry. This technique allows for rapid detection of these PFASs in human serum with limits of detection (LOD) in the low parts per billion range [16,20,21].

### 2.4. PFASs Detection Limits

The detection limits were constant for all the PFASs analytes in the data set (0.10 ng/mL). In the NHANES, two variables’ names were provided for each of these analytes. The value “0” meant that the result was at or above the limit of detection, “1” indicated that the result was below the limit of detection. For analytes with analytic results below the lower limit of detection, an imputed fill value was placed in the analyte results field. This value is the lower limit of detection divided by the square root of 2 (LLOD/sqrt(2)), which is 0.10/√2 = 0.07. As such, the LOD for each PFAS was either 0.10 or 0.07 [16,20,21]. A significant number of the participants tested for PFBS, PFHP, PFDO in our sample were below the LOD and thus these PFASs were excluded from the analysis.

### 2.5. Operationalizing Allostatic Load

Informed by prior studies [14], AL was operationalized by developing a cumulative index of physiologic dysfunction of the cardiovascular (systolic blood pressure—SBP, diastolic blood pressure—DBP, triglycerides, high-density lipoprotein (HDL) cholesterol, and total cholesterol), inflammatory (C-reactive protein—CRP), and the metabolic systems (body mass index—BMI, hemoglobin A1C, albumin, and creatinine clearance). Based on the distribution of AL markers in the dataset, the markers of interest were split into quartiles with the top quarter of their distribution considered to be high-risk for all markers apart from albumin, creatinine clearance, and HDL cholesterol, for which the bottom 25% of the distribution were considered to have the highest risk as determined by the literature [22,23,24,25,26,27,28]. Within the data, each participant in the study was assigned a value of 1 for those considered to be in the high-risk category or a value of 0 if for those in the low-risk category for all markers to add up to a total AL value out of 10. We dichotomized allostatic load scores as high if AL was ≥3 and low if AL was <3 [29,30].

### 2.6. Statistical Analysis

The dependent variable for this study was AL as a binary outcome (1 for high risk and 0 for low risk). The independent variables were the following PFASs: PFHS, PFDE, PFBS, PFHP, PFDO, PFNA, PFUA, PFOA, and PFOS. We performed Pearson’s correlation coefficient analysis and the variance inflation factor (VIF) test to check for multicollinearity. Due to the presence of multicollinearity, PFDE, PFNA, PFOS, PFUA, PFOA, PFHS were examined individually for their relationship with AL.

Descriptive statistics summarized the data within this study. In addition, logistic regression models were used to examine the associations between AL and each PFAS. Sociodemographic variables such as gender, age, and ethnicity were explored to see their distribution by PFASs and AL, how they affect the association between PFASs and AL, and subsequent adjustment for them in logistic regression modelling. Furthermore, behavioral variables including alcohol consumption, cigarette or tobacco use, and physical activity were also investigated to see their distribution in terms of PFASs and AL, how they affect the association between PFASs and AL, and subsequent adjustment for them in logistic regression modelling. These variables were adjusted for due to the literature indicating their relationship with AL and or PFASs [9,31,32,33].

To examine the associations between the response variable and the predictor variables, the Wald chi-squared *t*-test was used for categorical variables and the Wilcoxon *t*-test for numerical variables. In our analysis, to check the assumptions that PFASs variables were normally distributed, we performed a Kolmogorov–Smirnov Test (KS), which found that the PFASs were not normally distributed. Thus, the PFASs concentrations were log-transformed to satisfy normality assumptions. Considering the nonlinear exposure pattern of PFASs, the logistic regression analysis techniques used in this study offer better insight into the relationship between AL and PFASs as compared to studies that used linear analytic techniques.

All analyses were conducted using R version 4.1.2 (R Foundation for Statistical Computing, Vienna, Austria) with the RStudio platform version 9.1.372.

## 3. Results

**Figure 2:**
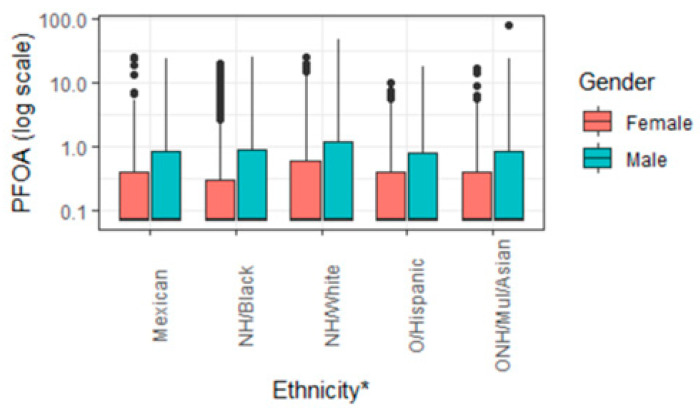
Distribution of PFOA by ethnicity and gender. * Note: Mexican American = Mexican, other Hispanic = O/Hispanic, non-Hispanic White = NH/White, non-Hispanic Black = NH/Black, other non-Hispanic race including non-Hispanic multiracial and Asian = ONH/Mul/Asian.

The total number of participants for this analysis was 23,482, with 51.88% being female. The mean age was 49.62 years. The ethnic composition of the study was as follows: 14.8 percent Hispanic Mexican American (*n* = 3480), 10.2 percent other Hispanic (*n* = 2384), 43.7 percent non-Hispanic White (*n* = 10,250), 21.2 percent non-Hispanic Black (*n* = 4981), and approximately 10.2 percent for other non-Hispanic races, including Asian and multiracial) (*n* = 2387). PFOA was differentially distributed by age, gender, and race/ethnicity (Figure 2). Prior studies using NHANES data have indicated the PFASs in this study are differentially distributed by race/ethnicity and gender [34,35].

Table 1 summarizes the ethnic composition of the study participants by gender. The composition was: Mexican American (49.3 percent males), other Hispanic (44.5 percent males), non-Hispanic White (49.3 percent were males), non-Hispanic Black (48.6 percent males), and non-Hispanic including multiracial and Asian (48.9 percent males). Table 2 summarize the variables by mean levels and standard deviation (SD) of the selected PFAS of interest and behavioral health variables (smoking, alcohol consumption, and physical activity).

Table 3 show the mean AL score by age category and ethnicity of participants in the study. AL was elevated for non-Hispanic Blacks in every age category. These variables include AL high ≥ 3 vs. low < 3 AL. AL levels increased with age. Older people had higher AL levels than other age groups as follows, non-Hispanic Black with a mean of 3.92, followed by other Hispanic ethnicity with a mean of 3.64, Mexican (mean 3.58), and other Hispanic with a mean of 3.47.

**Figure 3:**
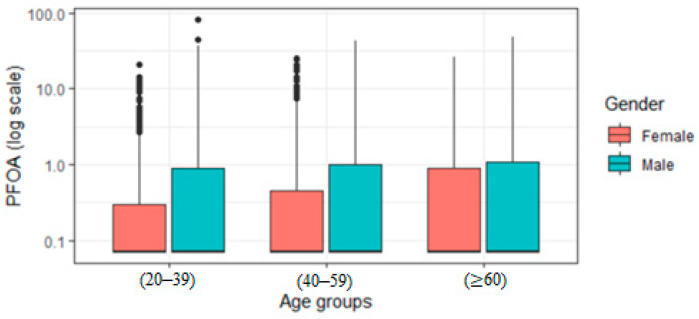
Distribution of PFOA by age group and gender.

Figure 2 presents the levels of PFOA for participants of different ethnicities and by gender and demonstrates that males have higher PFOA concentrations than females. Figure 3 reveals that PFOA is more elevated in those aged sixty and older for both females and males.

We ran separate models for each PFAS (unadjusted models) and used three different methods to check for multicollinearity:

**(a)**  **Correlation coefficient for PFAS variables (Table 4)**

Table 4 presents the Pearson correlation coefficients of the relationship between the selected PFAS with each other. PFAS were generally correlated with each other. A correlation of 0.4 and up presents concerns of multicollinearity. The analysis revealed that the PFAS have a correlation from moderate to strong which indicates the potential for multicollinearity.

**Figure 4:**
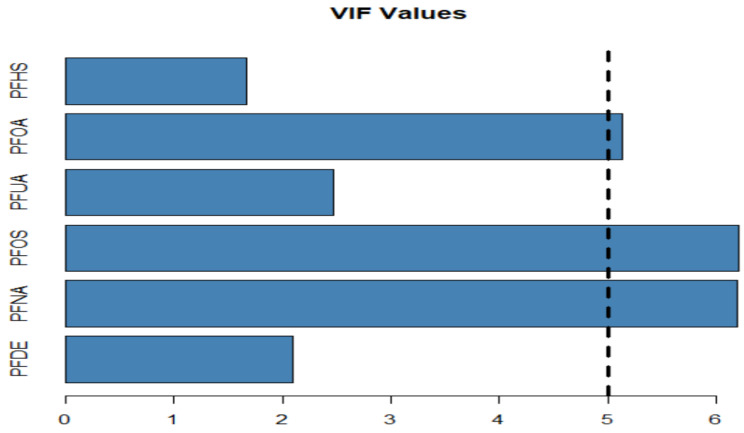
The variance inflation factor (VIF) values for selected PFAS.

**(b)**  **Variance Inflation Factor (VIF) test (Table 5)**
**(c)**  **Plotting the VIF to show to values of VIF for each PFAS (Figure 4)**

All three methods demonstrated multicollinearities between variables such as PFNA, PFOS, and PFOA with VIF values of 6.197, 6.212, and 5.139, respectively (Table 5). A VIF greater than five indicates a high/strong or potentially severe correlation, and thus the existence of multicollinearity.

As such, AL and each individual PFAS, adjusted for age, gender, ethnicity, physical activity, smoking, and alcohol consumption was used in the analysis.

Table 6 shows the association between PFASs and AL in the adjusted models. Each PFAS was entered into its own model and adjusted for the covariates of interest: age, gender, ethnicity, physical activity, smoking, and alcohol consumption. Logistic regressions modeling was used to investigate the association between AL each of the selected PFAS with covariates of interest included in the model. The results indicated a statistically significant positive association between AL and most of the PFASs, except PFUA, which was not statistically significant with a *p* value of 0.531.

Logistic regression modeling also found that AL was positively and significantly associated with age (*p*-value < 0.001, physical activity (*p*-value < 0.001) ethnicity (*p*-value < 0.001), alcohol consumption (*p*-value < 0.001), and smoking (*p*-value < 0.001).

The summary statistics of the behavioral health covariates for participants (Table 7) gives context to these findings by gender.

## 4. Discussion

This study explored the association between AL and PFASs. The major PFASs are environmentally and biologically persistent, with many having long half-lives in humans [36]. Allostatic load, an index of the chronic stress response, represents the physiological wear and tear on the body from extended exposure to stressful events. Markers of AL potentially shield the body in the short term and promote adaptation, but in the longer term, AL causes alterations in the body that bring forth disease. Indeed, AL has been shown to predict cardiovascular events and all-cause mortality, as it represents biological mediators in adaptation and maladaptation of the individual circumstances of life [37]. Our methods for calculating AL, which mirrored that of other studies [12,14,38,39] helped give insight into this understudied area.

Understanding the association between the cumulative physiological burden of stress (AL) and PFASs in the context of life’s demands, behavioral factors, and other factors [40] helps to close the gap in the literature on the role of environmental toxicants on stress and health.

This is especially the case, since people are exposed to stressors at all points in life with the cumulative burden altering health in the absence of resilience [41,42].

In our study, non-Hispanic Blacks had higher allostatic load scores across all age groups. This may be attributed to factors such as social and environmental exposures promoting the expression of genes more likely to put individuals in allostatic load. It may also be due to perceived racial discrimination or internalized racism [30].

This study found positive significant associations between AL and all the PFASs, apart from PFUA, in adjusted models evaluating AL and individual PFAS serum levels. In unadjusted models, AL was only significantly associated with PFOA *p*-value = 0.006.

This indicates that the perceived association between PFASs and AL is likely due to confounding by behavioral factors, where people who smoke or consume alcohol are likely to have elevated PFASs levels as well as a higher AL index.

Other studies’ [11,40] findings were similar to this study. They found an association between AL and health-damaging habits, such as smoking, low physical activity, and extensive alcohol consumption.

Males with higher serum PFOA had higher AL than females (Figure 2 and Figure 3). These differences may be explained by the divergent toxicokinetic of PFOA between the genders [43], the excretion pathway of females, such as menstruation and lactation [44], or its interaction with estrogen receptors [45]. That said, further analysis is needed by gender.

More research is needed to understand how PFASs contributes to AL. In addition, multicollinearity made analyzing all PFASs together impossible. Ultimately, behaviors such as physical exercise and limiting smoking and alcohol consumption can help to reduce AL. These behavioral changes, in addition to increasing resilience, can help promote longevity and decrease the likelihood of chronic diseases. If AL is unmanaged, the long-term effects of chronic, unmitigated, multi-year stress may be disease and subsequent death.

Limitations: This cross-sectional study depicts only a snapshot in time. A longitudinal study of the group may have yielded distinct results as people’s unique circumstances (finances, education, family, etc.) change resulting in people moving to different geographic areas, creating different social networks, gaining access to vital knowledge about PFASs exposure, and hence potentially decreasing exposure risk. In addition, due to the study design, it is unclear if PFASs serum levels increase AL or if AL makes once more likely to expose themselves to PFASs. In addition, multicollinearity made analyzing all PFASs together impossible: machine-learned models, Bayesian kernel machine regression, or weighted quantile sum (WQS) analysis may offer more insight into the interaction of multiple PFASs in promoting AL. Finally, a detailed examination of the associations by among different races/ethnicities and gender may have yielded different results.

## 5. Conclusions

AL levels in US adults are associated with PFASs serum levels. A reduction in alcohol consumption and smoking and increased physical activity may alter health outcomes. Future works should explore these findings in detail by gender and ethnicity and investigate the degree to which PFASs and other environmental and social exposures contribute to AL since AL is likely a mediator for several chronic diseases such as cardiovascular disease and cancer.

## Figures and Tables

**Table 1:** The composition and percentage of the ethnicity/race for the participants.

		Gender			
		Male		Female	
Ethnicity	Race	Number	Percent	Number	Percent
Hispanic	Mexican American	1715	49.3	1765	50.7
	Other Hispanic	1060	44.5	1324	55.5
Non-Hispanic	Non-Hispanic White	5052	49.3	5198	50.7
	Non-Hispanic Black	2419	48.6	2562	51.4
	* Other Race Including Multi-Racial	1168	48.9	1219	51.1
Total		11,414	48.12	12,068	51.88

* Non-Hispanic Asians were included in the “other race” category.

**Table 2:** PFASs mean and SD (standard deviation) by AL, and behavioral health.

	PFAS											
Variable	PFDE		PFNA		PFOS		PFUA		PFOA		PFHS	
AL	Mean	SD	Mean	SD	Mean	SD	Mean	SD	Mean	SD	Mean	SD
high	0.159	0.613	0.440	1.244	3.295	10.108	0.133	0.857	0.828	1.894	0.730	1.954
low	0.159	0.418	0.422	0.861	3.167	8.879	0.132	0.497	0.883	2.154	0.709	1.808
Physical Activity												
1 day	0.163	0.285	0.459	0.971	3.341	8.386	0.136	0.290	0.976	2.022	0.796	2.085
2 days	0.154	0.239	0.460	1.078	3.214	8.443	0.129	0.220	0.892	2.211	0.767	1.945
3 days	0.159	0.539	0.423	1.062	3.182	9.326	0.134	0.765	0.846	2.062	0.701	1.863
4 days	0.151	0.203	0.426	0.804	3.025	8.089	0.121	0.175	0.844	1.758	0.724	1.730
5 days	0.171	0.758	0.448	0.839	3.426	10.991	0.126	0.222	0.895	1.860	0.788	1.757
6 days	0.152	0.258	0.416	0.685	4.155	17.440	0.132	0.254	0.997	2.240	0.819	2.124
7 days	0.161	0.278	0.422	0.850	3.251	8.358	0.137	0.272	0.856	2.178	0.674	1.615
Smoking												
yes	0.156	0.375	0.441	0.900	3.476	10.214	0.129	0.556	0.926	2.040	0.782	1.891
no	0.161	0.581	0.420	1.112	3.016	8.660	0.136	0.728	0.813	2.071	0.668	1.844
Alcohol												
1 day	0.1626	0.2847	0.4591	0.9708	3.341	8.386	0.1358	0.2896	0.976	2.0225	0.7956	2.0852
2 days	0.154	0.2389	0.4599	1.0781	3.214	8.443	0.1291	0.2198	0.8924	2.2108	0.767	1.9453
3 days	0.1589	0.5388	0.4231	1.0619	3.182	9.326	0.1337	0.7649	0.8456	2.0616	0.7013	1.8627
4 days	0.1512	0.2028	0.4256	0.8036	3.025	8.089	0.1213	0.1745	0.8445	1.7576	0.7242	1.7302
5 days	0.1708	0.7576	0.4478	0.8389	3.426	10.99	0.126	0.2216	0.8952	1.8603	0.7876	1.7571
6 days	0.1521	0.2577	0.416	0.685	4.155	17.44	0.1324	0.2543	0.9971	2.2396	0.8193	2.1238
7 days	0.1611	0.278	0.4221	0.8499	3.251	8.358	0.1372	0.2722	0.8561	2.1779	0.674	1.6147

**Table 3:** AL’s score (≥, high and <3, low) and percentage by ethnicity and age group.

Age Group	Mean Score Allostatic Load	Percentage of 3 or More (High)	Percentage of Less Than 3 (Low)
**Age 20 to 39**			
Mexican	2.90	28.60	71.40
NH/Black	3.32	40.00	60.00
NH/White	2.66	22.70	77.30
O/Hispanic	2.69	23.40	76.60
ONH/Mul/Asian	2.63	19.70	80.30
**Age 40 to 59**			
Mexican	3.49	44.80	55.20
NH/Black	3.92	57.60	42.40
NH/White	3.26	38.50	61.50
O/Hispanic	3.47	43.30	56.70
ONH/Mul/Asian	3.08	32.60	67.40
**Age 60 and up**			
Mexican	3.58	47.40	52.60
NH/Black	3.83	56.00	44.00
NH/White	3.37	41.10	58.90
O/Hispanic	3.64	47.50	52.50
ONH/Mul/Asian	3.13	33.20	66.80

Note: Mexican American = Mexican, other Hispanic = O/Hispanic, non-Hispanic White = NH/White, non-Hispanic Black = NH/Black, other non-Hispanic race including non-Hispanic multiracial and Asian = ONH/Mul/Asian. PFOA was chosen as a representative PFAS to examine by gender, ethnicity, and age primarily because it was the most detected among the participants in the database.

**Table 4:** Correlation between PFASs variables.

PFASs						
	PFHS	PFDE	PFNA	PFUA	PFOA	PFOS
PFHS	1.000	0.196	0.404	0.112	0.516	0.539
PFDE	0.196	1.000	0.362	0.792	0.240	0.447
PFNA	0.404	0.362	1.000	0.260	0.542	0.566
PFUA	0.112	0.792	0.260	1.000	0.115	0.231
PFOA	0.516	0.240	0.542	0.115	1.000	0.674
PFOS	0.539	0.447	0.566	0.231	0.674	1.000

**Table 5:** Association between AL and individual PFASs of interest (unadjusted models) and variance inflation factor (VIF) values for each PFAS.

Variable	Coeff	SE	*p*-Value	(95% CI)		VIF
PFDE	0.082	0.068	0.234	−0.054	0.219	2.092
PFNA	−0.081	0.051	0.119	−0.184	0.021	6.197
PFOS	0.002	0.002	0.391	−0.002	0.125	6.212
PFUA	0.030	0.048	0.542	−0.067	0.126	2.467
PFOA	0.023	0.008	0.006	0.007	0.395	5.139
PFHS	0.002	0.002	0.391	−0.002	0.102	1.667

**Table 6:** Association between AL and PFASs of interest, behavioral health (physical activity, smoking, and alcohol consumption) age, gender, ethnicity (adjusted models *).

Variable	Coeff	SE	*p* Value
PFDE	−0.089	0.027	0.002
PFNA	−0.111	0.035	0.002
PFOS	0.005	0.002	0.032
PFUA	0.053	0.083	0.531
PFOA	0.027	0.008	0.002
PFHS	0.005	0.002	0.032

* Adjusted for age, gender, ethnicity, physical activity, smoking, and alcohol consumption.

**Table 7:** The composition and percentage of the behavioral health covariates for the participants.

		Gender			
		Male		Female	
Behavioral Health Covariates	Status	Number	Percent	Number	Percent
Alcohol	yes	9385	55.2	7605	44.8
	no	1630	28.7	4053	71.3
Smoking	yes	5986	58.6	4229	41.4
	no	5029	40.4	7429	59.6
Physical Activities					
1 day	At least ten minutes/day	842	61.5	528	49.5
2 days	At least ten minutes/day	878	49.5	896	50.5
3 days	At least ten minutes/day	7559	47.4	8393	52.6
4 days	At least ten minutes/day	420	44.9	515	55.1
5 days	At least ten minutes/day	572	47.5	632	52.5
6 days	At least ten minutes/day	153	48.3	164	51.7
7 days	At least ten minutes/day	591	52.7	530	47.3

## Data Availability

The NHANES dataset is publicly available online, accessible at cdc.gov/nchs/nhanes/index.htm (accessed on 12 February 2022).
